# Biomaterials for Alveolar Ridge Preservation as a Preoperative Procedure for Implant Treatment: History and Current Evidence

**DOI:** 10.3390/bioengineering10121376

**Published:** 2023-11-30

**Authors:** Tetsuya Sano, Ryutaro Kuraji, Yukihiro Miyashita, Kosei Yano, Dai Kawanabe, Yukihiro Numabe

**Affiliations:** 1 Department of Periodontology, The Nippon Dental University School of Life Dentistry at Tokyo, 1-9-20, Fujimi, Chiyoda-ku, Tokyo 1020071, Japan; t-sano@gem.hi-ho.ne.jp (T.S.); yukihiro.miyashita@tky.ndu.ac.jp (Y.M.); numabe-y@tky.ndu.ac.jp (Y.N.); 2 Heartful Dental Clinic, 4-12-3, Mejirodai, Hachioji-shi, Tokyo 1930833, Japan; 3 Lotus Dental Clinic, 3-13-11, Nishigotanda, Shinagawa-ku, Tokyo 1410031, Japan; yanokosei30@gmail.com; 4 Kawanabe Dental Clinic, T Building 1F, 4-21-4, Nishikojiya, Ota-ku, Tokyo 1440034, Japan; dai0806dk@gmail.com

**Keywords:** immediate implant placement, soft tissue grafting, absorbable alternative materials, open membrane technique, bone grafting materials, wound healing

## Abstract

In implant treatment, the reduction and structural changes in the alveolar ridge that occur after tooth extraction limit the length, width, and placement position of the implant body, impair esthetics, and, in some cases, make implant placement difficult. To solve these problems, an alveolar ridge preservation (ARP) technique, which is performed simultaneously with tooth extraction, generally aims to promote bone regeneration and prevent alveolar ridge reduction by filling the extraction socket with bone graft material and then covering it with a barrier membrane to protect against the invasion of epithelial tissue. The extraction socket provides a favorable environment for bone regeneration throughout the healing period because the blood supply is abundant, and it effectively retains the bone graft material by using the remaining bone wall of the socket. In recent years, advances in bioengineering technology have led to the development of graft materials with various biological properties, but there is currently no clear consensus regarding the selection of surgical techniques and materials depending on the condition of the alveolar ridge. This review will provide a comprehensive survey of the evidence accumulated to date on ARP, present many cases according to the clinical situation, and discuss various treatment options.

## 1. Introduction

After tooth extraction, the alveolar bone resorbs vertically and horizontally during the natural healing process [1]. Horizontal bone loss is usually greater than vertical bone loss, and it is more pronounced on the buccal bone than on the lingual and palatal sides of the alveolar ridge [2,3]. Most of the bone resorption occurs during the first 3 months after tooth extraction, followed by the dimensional loss continuing gradually over one year thereafter [4]. Since such morphological changes in the alveolar ridge limit the placement of implants into the ideal position [5], the alveolar ridge preservation (ARP) approach has been proposed to minimize the alveolar bone loss that occurs during the healing period after tooth extraction, thereby avoiding additional bone augmentation [6]. Compared to when left untreated after tooth extraction, ARP promotes host-derived bone formation by inhibiting epithelial invasion into the extraction socket [7]; therefore, it has been reported to be effective in reducing alveolar bone resorption [8].

The purpose of ARP, when first introduced, was to fill the extraction socket with bone graft material and then cover it with a coronally advanced gingival flap [9]. Subsequently, ARP was further developed with the aim of reducing vertical and horizontal physiological bone resorption after tooth loss by using a barrier membrane and bone grafting material together [10]. For ARP, various grafting materials are used mainly as scaffolds. In particular, autologous bone is considered the gold standard because it has three characteristics based on the concept of tissue engineering (osteogenic, osteoinductive, and osteoconductive ability), and it does not induce an immune response to antigen [11]. However, since ARP requires a relatively large amount of grafting material to fill the extraction socket, alternative materials such as allografts, xenografts, and alloplasts are generally used instead of autogenous bone, which is both surgically invasive and limited in volume [12,13].

The concept of using membranes in ARP is not only to prevent exposure and leakage of bone graft material from the extraction socket but also to prevent epithelial and connective tissue from invading the extraction socket based on the principles of guided tissue regeneration (GTR) techniques. When ARP was first proposed, an expanded polytetrafluoroethylene (e-PTFE) membrane, which was developed for the GTR procedure, was used, but the need for secondary surgery for membrane removal and the risk of bacterial infection due to membrane exposure were significant drawbacks [14,15]. Thus, when autologous soft tissue grafts, such as free gingival grafts and connective tissue grafts, began to be used, the invasiveness and insufficient supply associated with graft harvesting became an issue [16,17]. Subsequently, bioengineering technology has led to the active use of absorbable alternative grafting materials, such as collagen and synthetic polymer membranes, for ARP. In addition, open membrane techniques using dense polytetrafluoroethylene (d-PTFE) membranes with small pore size and low susceptibility to bacterial contamination have also been used [18] along with newly proposed biomaterials such as autologous dentin [19,20], platelet-rich plasma (PRP) [21], and platelet-rich fibrin (PRF) [22]; thus, there is a wide variety of graft and membrane combinations when selecting the technique for ARP.

A recent Cochrane review by Atieh et al. showed that ARP may be an effective approach to promote bone formation for future implant placement. In contrast, there were no clinically significant differences between grafting materials and membranes [23]. Therefore, there is currently no clear consensus regarding the selection of surgical techniques and materials depending on the condition of the tooth extraction socket and alveolar ridge, and it varies widely depending on the clinician’s and patient’s preferences, economic considerations, and cultural and educational background. Consequently, it is important to determine criteria for ARP based on scientific evidence as a guideline for determining treatment strategies, and ultimately, it is desirable to create a treatment decision tree. This review will provide a comprehensive survey of the evidence for ARP accumulated to date, provide a number of case studies for clinical situations, and discuss various treatment options.

## 2. Alveolar Ridge Changes following Tooth Extraction

When natural teeth are present, the jawbone is composed of the alveolar process and basal bone. Within the alveolar processes, an alveolar bone proper, that is, the tissue derived from the dental follicle, develops with tooth eruption. At the same time, it is gradually lost due to age-related change, as well as periodontitis and other peri-inflammation [24]. Furthermore, after tooth loss, the volume of the alveolar process is clearly reduced in both vertical and horizontal orientations [1]. Post-extraction resorption of the maxillary and mandibular alveolar processes is significantly greater buccally than lingually [3]. The decrease in width of the maxillary alveolar crest is greater than the decrease in height [25], and the change is maximal in the first month after tooth extraction [26].

A review article by Araujo et al. [27] evaluated the amount of hard and soft tissue changes in the alveolar crest up to 12 months after tooth extraction in humans. At 6 months after tooth extraction, the vertical dimensional reduction was 1.24 ± 0.11 mm on the buccal side, 0.84 ± 0.62 mm on the mesial side, and 0.80 ± 0.71 mm on the distal side, and the percentages were 11–22%. In contrast, horizontal reductions were larger, 3.79 ± 0.23 mm, and the percentages were 29–63%. These changes occurred rapidly in the first 3–6 months after tooth extraction and continued to decrease gradually thereafter. Namely, up to 50% of the original alveolar crest width is resorbed after tooth extraction, with most bone resorption occurring on the buccal side.

It has been suggested that the amount of tissue change after tooth extraction is influenced by the phenotype and the site of extraction. Chappuis et al. [28] investigated hard and soft tissue changes from immediately after extraction to the eighth week in maxillary anterior teeth, lateral incisors, and canine areas of patients who required single tooth extractions. The tooth extraction sites were divided into two groups according to the buccal bone thickness (≥1 mm: thick bone phenotype or <1 mm: thin bone phenotype, according to Chappuis et al. [29]), and the results were compared. Immediately after tooth extraction, the soft tissue thickness was not affected by bone phenotype (thick bone phenotype: 0.8 mm vs. thin bone phenotype: 0.7 mm). However, after an 8-week healing period, the soft tissue thickness in the thin bone phenotype area was 5.3 mm, approximately 7.5 times thicker than that immediately after extraction, although the soft tissue thickness in the thick bone phenotype area did not show any significant dimensional change. This may be due to the invasion of connective tissue, whose cell proliferation rate is faster than that of hard tissue, into the space made after hard tissue resorption. Indeed, the spontaneous soft tissue thickening observed in the thin bone phenotype resulted in a loss of only 1.6 mm of vertical soft tissue, compensating for the underlying 7.5 mm of vertical bone resorption. More than 51% of these soft tissue dimensional changes were observed within the first 2 weeks, whether the bone phenotype was thick or thin [28]. According to a study by Misawa et al. [30], when maxillary incisors and premolars were extracted alone, the amount of alveolar bone resorption varied by site, the smallest amount of vertical and horizontal change was observed in the lateral incisor area, and the largest amount of resorption was confirmed in the second premolar area.

**Figure 1:**
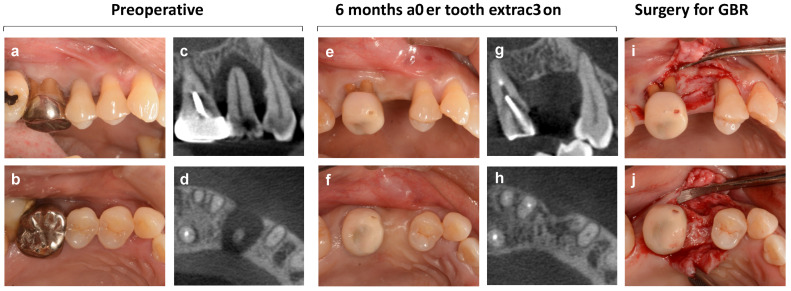
**Natural healing and changes in the alveolar ridge after tooth extraction.** (**a**,**b**) Intraoral photographs at the first visit. Maxillary right second premolar tooth (#15) is diagnosed as hopeless due to severe periodontitis, and then the tooth is extracted. Implant treatment was not initially planned. (**c**,**d**) Cone-beam CT images at the initial examination. (**e**,**f**) Intraoral photographs 6 months after tooth extraction. (**g**,**h**) Cone-beam CT images 6 months after tooth extraction. (**i**,**j**) Intraoral photographs during surgery of guided bone regeneration performed as pretreatment for implant treatment. The central portion of the alveolar ridge is cratered and depressed, and the buccal bone with malformed bony surface morphology is markedly reduced both horizontally and vertically.

In fact, as shown in Figure 1, even alveolar bone that was severely lost due to periodontitis showed a certain amount of bone formation during spontaneous healing after #15 extraction. However, the bone wall, especially on the buccal side, was horizontally and vertically depressed, and the bone surface healed, leaving an irregular morphology. On the contrary, the thickness of the soft tissue on the buccal side was greatly increased compared to the palatal side, and compensatory replacement of the buccal bone defect was observed.

## 3. Immediate Implant Placement after Tooth Extraction

Immediate extraction implant placement was first introduced by Schulte et al. [31] in 1978, and it has been used in clinical practice as an effective treatment [32,33]. Later, the idea that the alveolar bone morphology at the time of extraction can be maintained by placing an implant body in the fresh extraction socket was endorsed [34,35,36]. Araujo et al. [37] reported that, in an animal study using beagle dogs, they performed immediate implant placement to the extraction sites of the distal roots of the third and fourth premolars so that the gap that existed between the implant and the extraction socket bone wall at the time of surgery was filled with reticular bone 4 weeks later. The implant body surface was in contact with bone. During this period, the buccal and lingual bone walls underwent significant surface resorption, and the height diameter of the thin buccal bone wall decreased. As the healing process progressed, the buccal alveolar crest moved further towards the apex, more than 2 mm after 12 weeks.

Hard tissue alterations following immediate implant placement in humans were reported by Botticelli et al. [38]. Twenty-one implants were placed immediately after extraction in 18 patients, resulting in approximately 56% horizontal bone resorption in the buccal aspect and 30% in the lingual and palatal aspects at 4 months postoperatively. Similarly, Sanz et al. reported that single immediate implant placement after extraction of maxillary incisor teeth, canines, and premolars resulted in a large decrease in bone width on the buccolingual side, and immediate implant placement did not inhibit alveolar crest resorption after tooth extraction [39]. Similarly, many other studies suggest that immediate implant placement does not inhibit bone resorption after extraction [40,41,42,43].

To solve this problem, it is recommended that bone graft material be filled in the gap between the implant and extraction socket at the time of immediate implant placement. In a prospective study reported by Chen et al. [44], 30 patients scheduled for immediate placement of transmucosal implants in the maxillary anterior region were randomly assigned to one of the following groups: (i) no treatment at the time of immediate placement; (ii) filling bovine demineralized bone into the space between the implant and the bone wall; and (iii) filling bovine demineralized bone followed by closure of the extraction socket with a collagen membrane. At 4 years after surgery, the non-treatment group had 48.3% horizontal resorption of buccal bone, whereas the two grafted groups had a significantly lower resorption rate of 28–33%. These results indicate that filling the bone graft between the implant and the bone wall upon immediate implant placement reduces the amount of bone resorption compared with spontaneous healing, although it does not completely inhibit horizontal resorption; therefore, the use of bone filling in combination with immediate implant placement may have clinical significance as an ARP in the broadest sense.

In recent years, the concept of the “socket shield technique” has been proposed for implant placement immediately after tooth extraction, based on the hypothesis that resorption of the alveolar process can be prevented by leaving part of the tooth root after tooth extraction [1]. After hemisectioning the mandibular third and fourth premolars of a beagle dog, Hürzeler et al. cut the crown and further divided the distal root in the mesio-distal direction [2]. Then, after the lingual part of the divided distal tooth root was extracted, the remaining buccal tooth root was cut at a height of 1 mm from the buccal bone crest, and an implant was placed in the extraction socket immediately after the tooth extraction. The results of histological and clinical evaluations showed that the buccal bone was significantly preserved, and no resorption or inflammation of the alveolar process was observed. Only two randomized, controlled human trials have been reported [3,4], and their results showed that the socket shield technique promoted the maintenance of alveolar bone morphology, reduced the risk of marginal bone loss, and improved esthetics. However, in the latest systematic review [5], due to an insufficient number of studies, there were no consistent data to recommend the socket shield technique, and the author concluded that it cannot currently be recognized as an alternative treatment method.

### Clinical Case of Immediate Extraction Implant Placement with Bone Grafting

**Figure 2:**
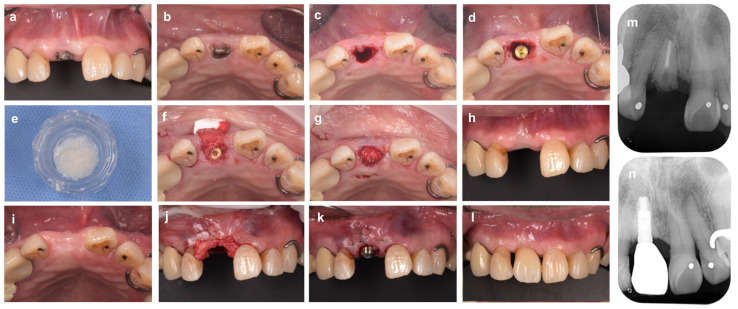
**Immediate implant placement after tooth extraction using bone graft material.** (**a**) At the first examination (labial aspect view). The maxillary right central incisor tooth (#11) is diagnosed as hopeless due to vertical root fracture. (**b**) Intraoral findings at the initial examination (occlusal aspect view). (**c**) Tooth #11 is carefully extracted to avoid damaging the surrounding tissue. (**d**) Immediate implant placement after tooth extraction. The implant body is placed in an ideal mesio-distal and labio-lingual position. A 2 mm gap is observed between the shoulder of the implant and the inner surface of the labial bone wall. (**e**) Bovine xenograft bone. (**f**) The xenograft is inserted into the gap and then covered with porcine collagen membrane. (**g**) In the labial and palatal sides, the membrane is secured to the gingival flap with 6-0 monofilament polypropylene sutures. (**h**,**i**) Labial and occlusal aspect views 6 months after implant placement. (**j**) A subepithelial connective tissue graft taken from the palate is applied to the gingival margin of the defect. (**k**) A connective tissue graft inserted under the gingival flap is fixed with sutures. (**l**) Intraoral findings (labial aspect view) 18 months after ARP (9 months after superstructure installation). (**m**) Preoperative X-ray image. (**n**) X-ray image at 18 months after ARP (9 months after superstructure installation).

As a representative clinical case (shown in Figure 2), the patient was a 70-year-old man with a collapsed crown on tooth #11. The tooth required extraction due to a vertical root fracture, and the patient desired esthetic improvement. Immediate implant placement after extraction was planned because the extraction socket consisted of a four-walled bone wall, including a thick labial bone wall (>1 mm), and only a mild vertical alveolar bone defect was observed. After the extraction of #11, the partial gingival flap elevation was performed using the tunnel technique without further incision. The gap between the bone wall and the implant was filled with deproteinized bovine xenograft bone (Bio-Oss, Geistlich Japan, Tokyo, Japan) to suppress the expected alveolar crest reduction. The entire extraction socket was then covered with an absorbable collagen membrane (Bio-Gide, Geistlich Japan, Tokyo, Japan) to prevent the leakage of the bone graft material. The buccal and palatal sides were stabilized with horizontal mattress sutures to secure the collagen membrane slipped under the gingiva flap. In this case, with high esthetic requirements, connective tissue grafting was performed 6 months after the immediate placement of the implants because additional soft tissue augmentation of the buccal gingival margin contour and interdental papillae was necessary. After another 2-month healing period, an abutment connection with the implant was performed, and a zirconia crown was finally placed on tooth #11. At the one-year postoperative follow-up, the gingival position was stable, and the esthetic outcome was satisfactory to the patient.

## 4. Alveolar Ridge Preservation

ARP aims to minimize the alveolar bone loss that occurs during the healing period after tooth extraction, thereby reducing the need for additional alveolar ridge augmentation at implant placement [6]. Hämmerle et al. [45] defined ARP as “preserving the ridge volume within the envelope existing at the time of extraction”, and they clearly distinguish it from ridge augmentation, defined as “increasing the ridge volume beyond the skeletal envelope existing at the time of extraction”. ARP was first proposed by Ashman et al. [46], in which the extraction socket was filled with bone graft material and covered with a gingival flap [9]. Subsequently, the predictability of the technique for ARP and its potential to minimize hard and soft tissue reduction in the alveolar crest after tooth extraction were reported [8,47,48], and this technique became more commonly performed. A recent network meta-analysis reported that the use of bone graft material is generally effective in reducing alveolar bone changes after tooth extraction [49].

### 4.1. Alveolar Ridge Preservation with Soft Tissue or Alternative Graft Materials

In both immediate implant placement and ARP, initial closure of the extraction socket was considered necessary to preserve and stabilize bone and soft tissue [17]. Sealing the extraction socket with a free gingival graft was first proposed by Landsberg [50,51] to obtain primary closure. However, the simple free gingival grafts, such as the so-called soft tissue punch technique [52], have a low success rate [50], which has been thought to be due to the fact that the blood supply to the graft can only be obtained from the aspect facing the extraction socket or from blood clots in the extraction socket [51]. To counter this disadvantage, Stimmelmayr et al. [53] presented a surgical technique called “Combination Epithelialized Subepithelial Connective Tissue Graft”. In this technique, the crestal area of the extraction socket was transferred to the hard palate and outlined. Then, a 1 mm deep incision was made perpendicular to the palatal surface to outline the epithelized component of the graft with a blade. This was followed by 1 mm deep mesial and distal horizontal relieving incisions to provide access to the subepithelial connective tissue portions. Through these relieving incisions, a split flap was raised toward the midline. Then, the anterior and posterior subepithelial tissue components were outlined with a blade using an incision straight through to the bone so that the combination epithelialized subepithelial connective tissue graft could be harvested without the periosteum with another split flap parallel to the palatal bone. In other words, this technique attempts to secure blood flow and improve the success rate by placing this connective tissue graft with palatal epithelium under the buccolingual mucosa in the extraction fossa [53].

Recently, a randomized, controlled trial comparing the ARP effect of collagen matrix xenografts with connective tissue grafts harvested via the procedure of Stimmelmayr et al. was published by Papace et al. [54]. All subjects had their extraction sockets filled with deproteinized bovine bone mineral containing 10% collagen (BioOss Collagen) and were then randomly assigned to the combined epithelialized connective tissue graft (CECG) group or the collagen matrix (CM) group, depending on the type of membrane material covering the extraction socket. However, the results showed no statistically significant difference in the amount of change in soft tissue thickness (CECG: 0.02 ± 0.66 mm vs. CM: 0.46 ± 0.89 mm) between the two groups [54]. In a randomized, controlled trial by Segnini et al. [55], after filling the extraction socket with deproteinized bovine bone graft, comparing an experimental group using collagen matrix xenograft (CMX) with a control group using connective tissue graft (FGG), they found no clinical difference between the two groups at 4 months postoperatively. Thus, similar outcomes can be expected with either material, but the use of soft tissue grafts may cause adverse events such as pain, gingival swelling, and discomfort at the harvest site, and it also increases operative time [56,57]. In addition, the texture and color of the grafted site may be inconsistent with the surrounding tissue, which is a significant esthetic drawback [58]. On the other hand, for ARP using absorbable collagen membrane, even if the membrane is completely covered by moving the buccal gingiva of the extraction socket coronally, a high probability of the subsequent exposure of the membrane (42–57%) has been reported [59]. The risk of the collapse and infection of the absorbable membrane is a concern. Therefore, the use of alternative soft tissue materials as the first choice for ARP is still open to consideration.

#### Clinical Cases of Alveolar Ridge Preservation Using Soft Tissue or Alternative Membrane Material

**Figure 3:**
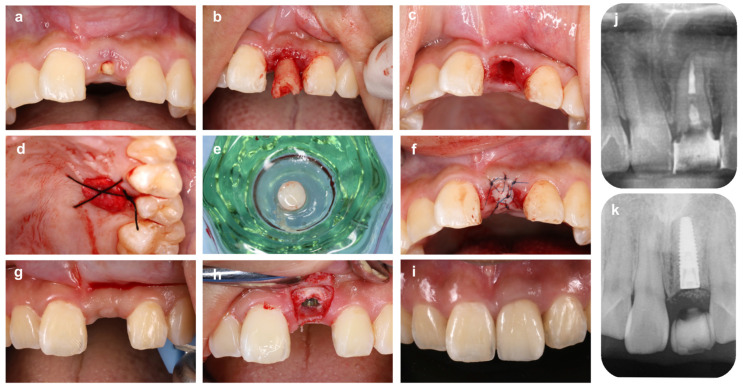
**Alveolar ridge preservation using free gum grafting and bone grafting material.** (**a**) On the first visit, a patient came to our hospital with a chief complaint of a fractured maxillary left central incisor tooth (#21). (**b**) Tooth #21 is carefully extracted to avoid damaging the surrounding tissue. (**c**) Extraction socket after granulation tissue removal. Although the labial alveolar bone wall remains, the apical bone is lost due to the periapical lesion. (**d**) The palatal wound surface from which the graft was harvested is protected by an absorbable collagen membrane. (**e**) A gingival punch is used to collect a round piece of free gingival graft from the palate. (**f**) After filling the extraction socket with bovine xenogeneic bone, free gingival grafting is performed to prevent gingival recession after tooth loss. (**g**) Four months after ARP: The height of the interdental papillae is maintained. (**h**) Implant placement is performed using a surgical guide. (**i**) Six months after placement of superstructure. The position of the papillae and the gingival margin line are symmetrical. Compared to before extraction, the soft tissue morphology is well maintained. (**j**) Preoperative X-ray image. Deep caries on the root surface and extensive bone resorption in the root apex are observed. (**k**) X-ray image 4 months after ARP (immediately after implant placement).

As shown in the case from Figure 3, in the esthetic zone, an option to prevent soft tissue reduction at the alveolar crest after tooth extraction is to prophylactically increase the tissue thickness by using autologous soft tissue grafts in place of the barrier membrane at the same time as ARP. In this case, a 25-year-old man was scheduled for implant treatment due to a crown fracture in tooth #21. The extraction socket was surrounded by four-walled bone, but significant bone and soft tissue reduction was predicted due to the thin buccolingual bone wall and the existing gingival inflammation. Therefore, after filling the extraction socket with deproteinized bovine xenograft bone (Bio-Oss, Geistlich Japan), the wound surface was closed with a free gingival graft taken from the palate by the punch technique [52]. At 6 months after ARP, the implant was placed, and a crown prosthesis with good esthetics of the #21 peri-mucosa was achieved without additional alveolar crest augmentation.

**Figure 4:**
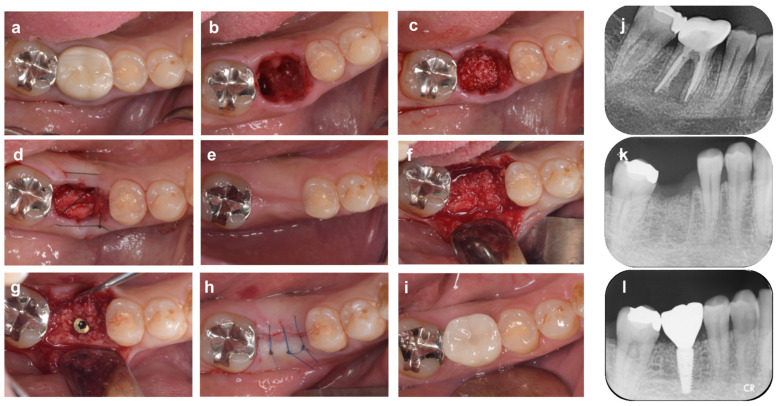
**Alveolar ridge preservation using an absorbable membrane and bone graft material**. (**a**) On the first visit, a patient came to our dental clinic with a chief complaint of spontaneous pain in the mandibular right first molar tooth (#46). (**b**) Tooth #46 was diagnosed as hopeless due to root fracture, and it was carefully extracted to avoid damaging the surrounding tissue. (**c**) Bovine xenogeneic bone is grafted into the tooth extraction socket. (**d**) The bone graft material and alveolar bone are covered by an absorbable membrane, and the gingival flap is stabilized by cross-mattress suture. (**e**) Four months after ARP: The width of the alveolar ridge has decreased slightly compared to before surgery. (**f**) Alveolar ridge bone surface at the time of implant placement. (**g**) Sufficient buccal bone thickness is maintained after implant placement. (**h**) After repositioning and suturing of the gingival flap. (**i**) Nine months after ARP (1 month after superstructure installation). (**j**) Preoperative radiograph. Extensive vertical bone resorption is observed from the furcation area to the root apex. (**k**) X-ray image immediately after ARP. The border between the xenograft and the alveolar bone is recognized. (**l**) Radiograph 9 months after ARP (1 month after superstructure installation).

Conversely, when high esthetics are not required, as in the case of molars, ARP can be performed with a simpler technique by using an alternative material as a barrier membrane. In the case of Figure 4, the patient (39-year-old man) came to the clinic with a chief complaint of spontaneous pain in tooth #46. Since #46 could not be saved due to root fracture, the tooth was extracted with as little tissue damage as possible, and the granulation tissue was carefully removed from the extraction socket. Reduction of alveolar bone was observed in the center of the buccal bone wall and the alveolar septum. After filling the extraction socket with bovine heterogeneous bone (Bio-Oss, Geistlich Japan), an absorbable collagen membrane (Bio-Gide, Geistlich Japan) was used to cover the bone graft and alveolar bone surface, and the gingival flap was stabilized with cross-mattress sutures. To reduce postoperative discomfort, the gingival flap was not moved coronally, a part of the collagen membrane was left exposed in the oral cavity, and the extraction socket was maintained in an open wound (for the open membrane approach, see Section 5). Four months after ARP, the extraction socket was filled with a sufficient amount of new bone for implant placement, although the width of the alveolar crest was slightly reduced compared to preoperatively.

**Figure 5:**
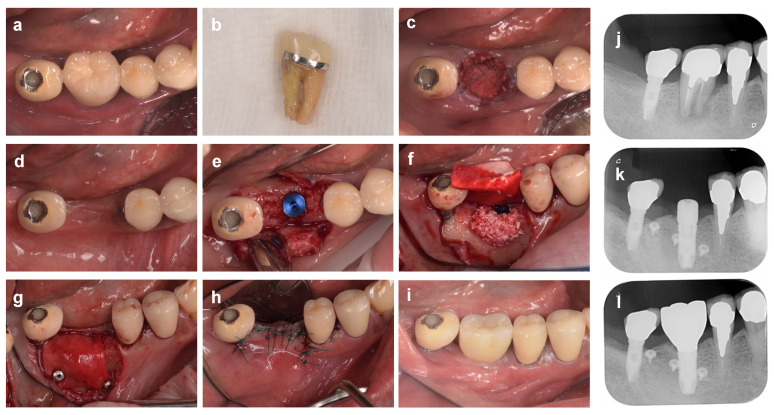
Alveolar ridge preservation using a resorbable membrane and bone grafting material, followed by guided bone regeneration. (**a**) On the first visit, a patient came to our clinic with a chief complaint of spontaneous pain in the mandibular right first molar tooth (#46). (**b**) Tooth #46 is extracted due to refractory periapical periodontitis. (**c**) The xenografted bovine bone is filled into the extraction socket and then covered with an absorbable membrane, and the gingival flap is stabilized with cross-mattress sutures. (**d**) Four months after ARP. A depression of the alveolar ridge is observed on the buccal side. (**e**) Alveolar ridge bone surface at the time of implant placement. The buccal bones are reduced both vertically and horizontally. (**f**) A mixed graft of autologous and xenograft bone is placed on the buccal side. (**g**) The graft material and bone surface are covered with a porcine collagen membrane, and then the membrane is fixed with titanium screw pins. (**h**) After repositioning and suturing of the gingival flap. (**i**) Eighteen months after ARP (6 months after superstructure installation). (**j**) Preoperative radiographs. (**k**) Radiograph 10 months after ARP. (**l**) Radiograph 18 months after ARP (6 months after superstructure installation).

Next, in the case shown in Figure 5 (69-year-old man), #46 was extracted because of severe mobility and occlusal pain due to refractory apical periodontitis. No incision was made into the alveolar crest, and a partial gingival flap elevation was performed. The buccal bone wall was destroyed by the periapical periodontitis and wide cleft. After filling the extraction socket with bovine heterologous bone (Bio-Oss, Geistlich Japan), the bone graft material and alveolar bone were covered with an absorbable collagen membrane (Bio-Gide, Geistlich Japan), and the gingival flap was stabilized with cross-mattress sutures. As predicted in advance, the buccal alveolar bone was vertically and horizontally resorbed at the time of implant placement four months after ARP, so a guided bone regeneration (GBR) technique was performed using a combination of heterologous bone and collagen membrane. In both cases shown in Figure 4 and Figure 5, the morphology of the alveolar ridge remained stable and in harmony with the superstructure during the follow-up periods of 12 and 18 months after surgery, respectively.

### 4.2. Alveolar Ridge Preservation with Non-Absorbable d-PTFE Membrane

Before soft tissue sealing was reported, the technique of sealing the extraction socket with an expanded polytetrafluoroethylene (e-PTFE) membrane was used for the immediate placement of implants. Lazzara et al. [14] hypothesized that epithelial tissue invasion inhibited healing because the extraction socket was open at the time of immediate implant placement and that sealing the extraction socket with the e-PTFE membrane would allow bone tissue regeneration around the implant, similar to the acquisition of new attachments by the GTR technique. In the original concept, leaving the e-PTFE membrane exposed during the healing period was considered acceptable [14], but later, Becker et al. published a method in which the extraction socket is completely closed by a rotated pedicle flap after placement of the e-PTFE membrane over the implant body at the time of immediate implant placement to prevent membrane exposure, thereby reducing the risk of postoperative infection and simultaneously promoting bone formation [15]. However, the complete closure of the extraction socket requires complex techniques such as a periosteal-releasing incision and coronally advanced flap due to the lack of soft tissue around the extraction socket [15]. Another disadvantage of ARP using the e-PTFE membrane is the need for secondary surgery for membrane removal and the high possibility of membrane exposure (30%) during the healing period after tooth extraction [16,60,61]. In fact, Simon et al. [61] compared the results of placing implants in extraction sockets between the e-PTFE membrane exposed in the oral cavity and the membrane completely covered, and they found that membrane exposure allowed bacteria to penetrate to the inner surface of the membrane and significantly reduced the rate of osteogenesis (96.6% vs. 41.6%). Therefore, given that e-PTFE membranes were not developed for ARP in extraction sockets [62], their use would not be recommended during ARP procedures today.

In contrast, ARP using dense polytetrafluoroethylene (d-PTFE) membranes, as presented by Bartee [18,63], improves on the drawbacks of the e-PTFE membrane technique described above. Namely, the high density and small pore size (0.2 μm) of the d-PTFE membrane are thought to protect the bone graft and implant body under the d-PTFE membrane by providing physical protection against bacterial contamination, thereby eliminating the need for primary closure of the extraction socket [18,63]. Recently, Chatzopoulos et al. [6] reported a systematic review of ARP with d-PTFE membranes. A meta-analysis showed that the combined use of allograft and d-PTFE membranes in the extraction socket significantly increased the keratinized mucosa width by an average of 3.49 mm compared to leaving the tooth untreated after extraction. They also reported that there was no difference in the horizontal radiographic volume change of alveolar bone in the d-PTFE group compared to the untreated group, but the vertical change was significantly less by an average of 1.06 mm [6].

According to a randomized, single-blind, comparative study by Arbab et al. [64], when using a barrier membrane in combination with bone graft material for ARP, there were no statistically significant differences in horizontal alveolar crest width and vertical alveolar crest height between the d-PTFE membrane group and the absorbable collagen membrane group. Bone tissue biopsied from sites treated with ARP using a trephine bar at the time of implant placement 4 months after extraction showed no histological differences between the d-PTFE and absorbable collagen membranes [64]. In summary, considering the high frequency of membrane exposure after primary closure and the infection risk when using absorbable membranes, ARP using the open membrane technique with d-PTFE nonabsorbable membranes has advantages in terms of technical simplicity and infection protection. In addition, compared to the absorbable membranes, d-PTFE membranes are stiffer and less deformable, and they may be more effective for space-making in ARP extraction sockets with large bone wall defects. Interestingly, Sun et al. reported that in an extraction socket with more than 3 mm of labial bone wall loss, ARP with a combination of d-PTFE membrane and freeze-dried irradiated allogenic bone inhibited reduction in alveolar ridge width of approximately 1.6 mm within 1 mm of the alveolar crest compared to the untreated site [65]. However, at present, there are few clinical studies comparing the effects of the bioabsorbable membrane and d-PTFE membrane, and there seems to be no clear evidence to help choose a technique for ARP.

#### Clinical Cases of Alveolar Ridge Preservation with the Open Membrane Approach Using Non-Absorbable Membranes

**Figure 6:**
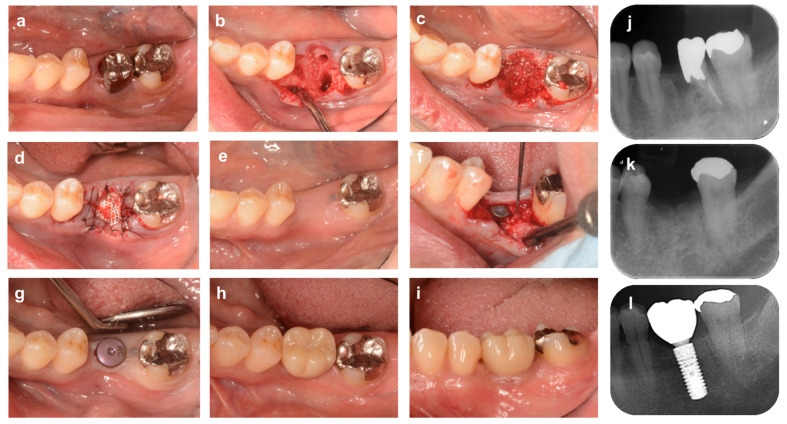
**Alveolar ridge preservation using a non-absorbable membrane and bone graft material (molar area).** (**a**) At the time of the first visit. A patient came to our clinic complaining of gingival bleeding at the distal root of the lower left first molar (#36) and discomfort during occlusion. (**b**) The distal root of tooth # 36 has bony adhesion, but the tooth is carefully extracted to avoid damaging the surrounding tissue. The mesio-buccal part of the alveolar crest is strongly resorbed horizontally, and the center of the alveolar ridge is depressed. (**c**) The extraction socket and the depression near the alveolar crest are filled with bovine xenogeneic bone. (**d**) The bone material and bone surface are covered with an absorbable collagen membrane, and the flap is stabilized with cross-mattress sutures. (**e**) Four months after ARP. (**f**) Alveolar ridge bone surface at the time of implant placement. (**g**) After healing, abutment placement in secondary surgery. (**h**,**i**) Occlusal and buccal views 18 months after ARP (6 months after superstructure placement). (**j**) Preoperative X-ray image. (**k**) X-ray image 4 months after ARP. (**l**) X-ray image 18 months after ARP (6 months after superstructure installation).

Figure 6 shows a case of the open membrane approach with a d-PTFE membrane in a molar (54-year-old man). Tooth #36 had been treated by a different dentist with hemisection due to a class III furcation lesion, and the mesial root had already been extracted at the time of his visit to our clinic, but the remaining distal root had a 6 mm deep residual periodontal pocket with bleeding on probing. We extracted #36 due to the patient’s chief complaint of bleeding during brushing and severe discomfort during occlusion. The buccal alveolar bone of the mesial site was strongly resorbed horizontally, and the center of the alveolar crest was depressed. The extraction socket of the distal root consisted of a four-bone wall, but the buccal bone wall was thin, less than 1 mm, and this case was judged to be an indication for ARP. The gingival flap was largely formed with a full-thickness flap to ascertain the alveolar bone morphology and ensure membrane coverage. After filling the extraction socket and the mesial alveolar crest depression with bovine heterogeneous bone (Bio-Oss, Geistlich Japan), the bone graft material and alveolar bone were covered with d-PTFE membrane (Cytoplast, Osteogenics, Lubbock, TX, USA) and the gingival flap was stabilized with cross-mattress sutures. Taking advantage of the d-PTFE membrane’s ability to resist bacterial contamination, the extraction socket was maintained in an open wound with the membrane exposed. One month after ARP, the d-PTFE membrane was removed. At the time of implant placement, 4 months post-operation, the width of the alveolar bone crest was slightly reduced compared to preoperatively, but the sufficient height and width of the alveolar ridge were preserved for implant placement.

**Figure 7:**
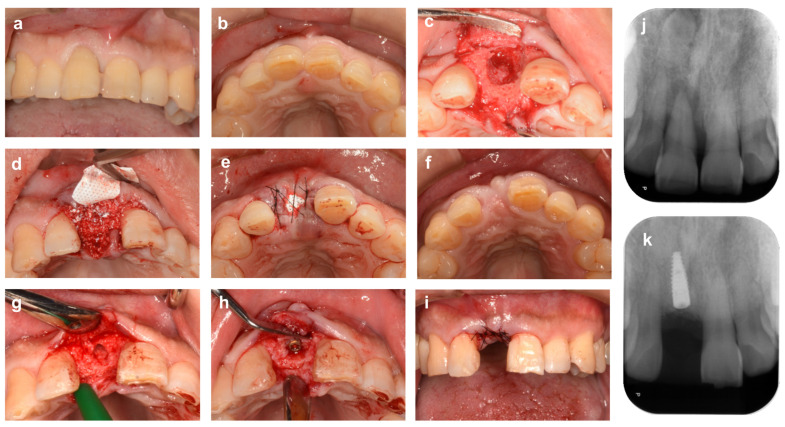
**Alveolar ridge preservation using a non-absorbable membrane and bone graft material (anterior tooth area).** (**a**,**b**) Labial and occlusal aspect views at initial examination. A patient came to our clinic with a chief complaint of severe movement of the maxillary right central incisor tooth (#11). (**c**) Tooth # 11 was diagnosed as hopeless due to severe periodontitis and extracted. The buccal bone wall of the extraction socket is extensively lost. (**d**) The extraction socket is filled with bovine xenogeneic bone. (**e**) The bone material and bone surface are covered with a d-PTFE membrane, and the gingival flap is stabilized with cross-mattress sutures. (**f**) Six months after ARP. (**g**) Alveolar ridge bone surface after socket formation for implant placement. (**h**) After implant placement. Sufficient thickness of labial bone is ensured. (**i**) After repositioning and suturing the gingival flap. (**j**) Preoperative X-ray image. (**k**) X-ray image 6 months after ARP (immediately after implant placement).

Figure 7, on the other hand, shows a case of ARP performed on a maxillary anterior tooth (48-year-old man). Tooth #11 showed severe alveolar bone loss due to periodontitis, especially in the extensive cleavage of the labial bone wall extending to the root apex. After extraction of tooth #11, the extraction socket was filled with bovine heterogeneous bone (Bio-Oss, Geistlich Japan) and then covered with a d-PTFE membrane (Cytoplast, Osteogenics). The technique using d-PTFE membrane, in this case, is basically based on the same concept as in the above-mentioned case in the molar region (Figure 6), but it was chosen for greater efficacy in preserving the alveolar ridge with defects in the labial buccal wall, as suggested by Sun et al. [65]. At the implant placement at 6 months postoperatively, horizontal and vertical resorption of the labial side of the alveolar ridge was observed, but there was sufficient bone volume for implant placement without additional bone augmentation.

## 5. The Need for Primary Closure of Extraction Sockets by Gingival Flaps

The need for wound closure of the extraction socket in the ARP technique has been debated, but it should be noted that the concept is slightly different from that of alveolar ridge augmentation such as GBR [66]. That is, GBR induces bone regeneration in the alveolar crest where wound healing is already finished, whereas ARP provides a more favorable environment for bone regeneration in the extraction socket where blood flow is abundant and active healing is ongoing. In addition, because the extraction socket is mostly composed of three to four bone walls, retention of bone graft material and space-making is easier than in GBR. Therefore, it has become more common in recent years to believe that complete wound closure by an advanced flap is unnecessary in ARP and that the barrier membrane can be exposed.

In a randomized, single-blind clinical trial by Engler-Hamm et al. [67], hard and soft tissue changes were compared in a split-mouth design between a control group with primary soft tissue closure and a test group with exposed membrane. At 6 months after ARP with a bone graft and co-polymer bioabsorbable membrane, there was no significant difference in bone width between the two groups. However, postoperative discomfort was significantly lower in the test group than in the control group. Furthermore, the position of the muco-gingival junction was significantly more displaced toward the crown in the control group than in the test group, with a mean difference of approximately 2.5 mm. Barone et al. [68] also randomized and allocated subjects to a control group (full-thickness mucoperiosteal flap and primary soft tissue closure) or a test group (flapless surgery to expose the membrane) in ARP using porcine bone and collagen membrane. The analysis results of bone core samples taken during the implant placement 3 months later showed no significant differences between the two groups in histomorphometric findings, such as percentage of newly formed bone, graft remaining rate, and ratio of bone marrow cavity.

A systematic review by Martins et al. [69] compared various techniques for sealing the extraction socket during ARP and performed a meta-regression and network meta-analysis to integrate these articles. The results showed that ARP with complete closure of the wound by a coronally advanced flap (CAF) was most likely to preserve the alveolar ridge. However, when compared to the open membrane technique with absorbable or nonabsorbable membranes, the additional preservation of alveolar ridge width by the coronally advanced flap was approximately 0.08 mm, suggesting little clinical benefit. ARP with the coronally advanced flap is less costly because there is no need for an alternative membrane, but it is also more technically challenging because it requires a periosteal releasing incision and can cause postoperative swelling, pain, the gingival recession of adjacent teeth, and decreased keratinized mucosa width [68]. Therefore, leaving the biomaterial exposed to the oral cavity during ARP without complete closure of the wound surface may be an effective suggestion to significantly reduce surgical complexity, time, and adverse events. On this point, the review article by Martin et al. [69] included four clinical studies of ARP using only bone graft materials without membrane coverage, three of which (Brkovic et al. [70]; Lim et al. [71]; Saito et al. [72]) showed localized alveolar ridge resorption, whereas the remaining study (Jung et al. [73]) showed resorption of 77.5% of the alveolar ridge width. Based on these results, the authors state that ARP using only biomaterials without a barrier membrane is not recommended.

Based on this accumulated evidence, there does not seem to be any clinical significance of primary closure by a coronally advanced flap in ARP. However, since exposure of the bone graft material filled in the extraction socket may reduce the effectiveness of ARP, it is currently desirable to cover the bone graft with a barrier membrane.

## 6. Bone Grafts for Alveolar Ridge Preservation

### 6.1. Biomaterials Science of Bone Graft Materials

In the standard ARP technique, the use of a barrier membrane prevents invasion of soft tissue such as epithelium and connective tissue into the extraction socket, and the bone graft material provides mechanical support and serves as a scaffold and lattice for surrounding cells to infiltrate and migrate through the graft [74]. The bone graft material serves primarily as a scaffold for cellular growth involved in osteogenesis and performs a supportive role in assisting space-making to maintain the shape of the barrier membrane. Bone-grafting materials used in the maxillofacial region can be classified into autografts (autogenous bone) and allografts (allogeneic bone) for human-derived bone, xenografts (heterologous bone) for animal-derived bone as non-human source materials, and alloplasts (artificial bone and materials) [11].

Autografts are taken from the same donor site and transplanted to a different site. Of the various bone grafting materials, the autograft is the only one that possesses all three elements of tissue engineering, namely an osteogenic ability (stem cells), osteoinductive ability (growth factors), and osteoconductive ability (scaffolds), and it is considered the “gold standard” of bone grafting materials [75]. However, although donor sites exist both inside and outside the oral cavity, there is the disadvantage of limiting the amount collected and the requirement for additional invasive procedures [13]. On the other hand, an allograft is a tissue that is not autologous from another human and does not have the volume limitations that are the drawback of an autograft [76]. Allografts are used regularly for bone regeneration with minimal risk of disease transmission through screening, tissue processing, and virus eradication. However, the risk that current methods cannot eliminate the possibility of tissue contamination or disease transmission by new unidentified pathogens still remains. Allografts are differentiated by processing method into fresh-frozen bone allograft (FFB), freeze-dried bone allograft (FDBA), and demineralized freeze-dried bone allograft (DFDBA). The FFB has the highest osteoconductive and osteoinductive potential of all available graft materials [77], but it is currently not used because of the risk of disease transmission. The FDBA, in which the human leukocyte antigenicity on the graft particle surface is reduced by the freeze-drying process [78], has osteoinductive and osteoconductive potential [79]. The DFDBA is a demineralized bone graft with a rapid resorption rate [80], and it often has not only osteoconductive but also osteoinductive potential due to growth factors such as bone morphogenetic proteins (BMPs) remaining in the graft material [81].

Xenografts are grafted tissues derived from species other than humans, i.e., animals, typically porcine or bovine bone mineral, with hydroxyapatite (HA) as the primary inorganic phase [82]. Thermal and chemical processes can reduce antigenicity to negligible levels but, at the same time, remove osteomorphic potential, usually leaving only osteoconductive potential [83]. Like xenografts, alloplasts have osteoconductive potential and have no osteogenic and osteoinductive potential of their own [84]. The most commonly used alloplasts are HA, tricalcium phosphate (TCP), and bioactive glass. HA is a calcium phosphate-based biomaterial with a composition and structure similar to natural bone minerals [85], and its absorption rate depends on the formation method, composition, and structure of the ceramic [86]. HA synthesized at high temperatures is dense, and absorption is limited [87]; however, when synthesized at low temperatures, it is porous and slowly absorbed [88]. TCP is divided into two categories: α-TCP and β-TCP. β-TCP, in particular, shows good biocompatibility and osteoconductive capacity and is used as a partially resorbable filler that allows replacement with new bone [84]. Bioactive glass is composed of silicon dioxide, calcium oxide, sodium oxide, and phosphorus pentoxide [89], and it precipitates hydroxyapatite in aqueous solution [90]. Nanoparticles of bioactive glass have the ability to bind to hard and soft tissues without rejection, the ability to induce cementoblast proliferation has been reported by in vivo studies [91], and bone formation in close contact with the particles has been reported in clinical studies [89].

Furthermore, the clinical efficacy of autologous dentin [19,20], platelet-rich plasma (PRP) [21], platelet-rich fibrin (PRF) [22], enamel matrix derivative [92,93], and hyaluronic acid gel [94] as new possible grafting materials or as adjunctive materials have recently been investigated.

### 6.2. Scientific Considerations for Bone Grafts Based on Clinical Studies

To review the effect of different bone grafts for ARP, we searched for studies with a low risk of bias among randomized, controlled trials that included a test group of various bone grafts filled into the extraction socket and a control group with extraction alone with no treatment. In studies using xenografts [95,96], porcine bone in combination with collagen membrane was used in the test group, whereas the control group underwent tooth extraction alone.

Barone et al. [95] found no significant differences in clinical parameters over a 3-year observation period, whereas Festa et al. [96] showed that, during the initial healing period of 6 months after treatment, the use of bone graft significantly reduced horizontal bone resorption by 1.9 mm and vertical bone resorption at the center site by 1.9–2.5 mm. Similarly, in a study comparing a test group using a combination of bovine bone and collagen membrane with an untreated group, Cha et al. [97] reported that significant vertical and horizontal bone resorption was approximately 3 mm and 2 mm, respectively. However, a similar study by Iorio-Siciliano et al. [98] showed no significant difference.

Regarding comparisons between different graft materials, the ARP effects of DFDBA or bovine xenogeneic bone in combination with various membranes for 6 months have been evaluated. The results showed that allograft significantly reduced bone resorption by 1.0 mm more than bovine bone graft in the study by Santana et al. [99], whereas the studies by Scheyer et al. [100] and Serrano Mendez et al. [101] showed no significant differences between the two materials. In addition, a retrospective study by Chisci et al. [102], comparing the combination effects of bovine bone and collagen membrane or autologous bone and collagen membrane, found no statistically significant difference between the two groups.

Regarding alloplasts, β-TCP and hydroxyapatite are currently being used for ARP [103]. In particular, plastic materials containing collagen, such as β-TCP/type I collagen cones, have the advantage that they can be used alone without a barrier membrane because the graft material is less likely to leak out of the extraction socket. However, the majority of studies reported to date on the superiority of alloplasts for ARP have various problems, as follows: lack of information on the amount of change in bone resorption; failure to include an untreated post-extraction group as a control group; lack of consistency in the type of barrier membrane between groups; and the complexity of study designs due to the combined use of materials, etc. [12,72,104,105,106]. These make it difficult to properly evaluate the clinical efficacy of using individual alloplasts.

A systematic review by Atieh et al. [23] also reported a similar trend in their meta-analysis. In comparing the xenograft group to the untreated group, integrated data from a total of 184 patients included in six randomized, controlled trials showed that the xenografts suppressed an average of 1.18 mm of horizontal bone resorption and 1.35 mm of vertical bone resorption. For alloplasts, a polylactide and polyglycolide (PLA-PGA) sponge group and the untreated group were compared in only one randomized, controlled trial, and alloplasts reduced the vertical bone resorption by 3.73 mm, but there was no description of horizontal changes. Furthermore, when a total of 87 patients included in three randomized, controlled trials were synthesized in a meta-analysis to compare xenografts and allografts, allografts had a 0.40 mm horizontal and 0.45 mm vertical advantage in reducing bone resorption. However, regardless of the type of grafting material, certainty of the evidence was rated very low for both outcomes because of the unclear risk of bias, high heterogeneity, and uncertainty due to a single study; therefore, the authors concluded that ARP may minimize overall changes in the alveolar crest, but the evidence is very uncertain.

### 6.3. Criteria for the Use of Bone Grafts in Alveolar Ridge Preservation

As noted above, the reason for the wide variation in ARP efficacy between studies may have been influenced by the condition of the extraction socket studied, the host’s general condition, and environmental factors rather than by the characteristics of the grafting material [95,96,97,98]. However, many articles do not provide clear criteria for selecting sites for ARP, which severely limits the interpretation of the current evidence to determine the indications for ARP. In considering this point, the decision tree for the selection of bone graft, membrane, and regenerative materials for periodontal tissue regeneration in natural teeth, as suggested by Cortellini et al. [107], is instructive. Namely, depending on the bone morphology of the defect, this clinical guideline recommends the use of no graft material for a “containing defect” consisting of a three-bone wall and a combination of bone graft material and barrier membrane for a “non-containing defect” with partial lack of a bone wall. Of course, the healing process for extraction sockets that do not achieve primary closure by soft tissue is different than that for intrabony defects in periodontal tissue. However, this criterion for treatment decisions seems to make sense, stating that ARP is not always necessary in four-walled extraction sockets where a blood clot can be maintained, whereas the use of bone graft material is preferable in extraction sockets requiring space-making due to wide bone loss.

It has also been reported that the bone graft materials filled at ARP interfere with the normal healing of the extraction socket [108], and particles of bone graft can remain in the socket for more than 6 months [109]. In a systematic review by De Risi et al. [47], the percentage of graft particles remaining 3 to 7 months after tooth extraction was lowest for allografts (12.4–21.11%). In contrast, at 7 months, xenografts (37.14%) and alloplasts (37.23%) had the highest percentage. However, there was no statistically significant difference in the ratio of bone to connective tissue when bone grafts were used compared to those that were untreated post-extraction.

Taken together, these studies clearly showed that bone grafting on ARP reduced alveolar bone resorption compared to leaving a tooth untreated after extraction. In contrast, there was little evidence of a difference in the clinical effect of different materials. Realistically, however, almost all studies have used a barrier membrane in conjunction with the bone graft material because of concerns that the graft material would leak into the oral cavity if only the bone graft material were used without the barrier membrane. Thus, it is difficult to evaluate the effect of the grafting material alone. Another factor complicating research in this area may be that a wide variety of graft material and barrier membrane combinations are selected at the discretion of the operators in the clinical setting in which ARP is performed. For these reasons, larger, rigorously designed studies are needed to evaluate the effects of the graft material itself in ARP and to reach a consistent consensus.

## 7. Potential Risk Factors That Affect the Clinical Outcomes of Alveolar Ridge Preservation

Potential risk factors for poor clinical outcomes in ARP include local factors such as bacterial infection and systemic factors such as smoking and diabetes mellitus, although the associations between these factors and ARP have not been studied at all. Therefore, when judging the prognosis of surgical procedures for ARP, it is advisable to refer to several research reports on periodontal tissue regeneration therapies such as GTR and enamel matrix derivatives (EMD), as well as GBR procedures.

A bacterial infection of the barrier membrane may cause impaired wound healing and unpleasant adverse events after periodontal tissue regeneration [1]. Sander and Karring [2] showed that bacterial accumulation due to membrane exposure reduced the level of new bone formation. Furthermore, Machtei et al. [3] evaluated the effect of early membrane exposure on the outcome of regenerative therapy with GTR and GBR in a systematic review. The results of this meta-analysis showed that membrane exposure during healing had only a marginal effect on attachment gain to GTR around natural teeth, but it significantly reduced the amount of new bone formation by GBR around implants. Therefore, it is generally recognized that the main local factor affecting bone formation is bacterial colonization of the membrane material and that effective control of bacterial contamination is a major point in the regeneration process [4,5]. Therefore, it is expected that antibiotics and disinfectants should be used in combination with ARP and GBR postoperatively to prevent postoperative infection in implant treatment. Still, these recommendations are often based on personal experience, and there is currently little evidence available. Two randomized, controlled clinical trials [6,7] found no significant benefit from systemic antibiotic use in standard oral implant treatment regarding perioperative postoperative infections and complications. Even in GTR around natural teeth, the use of systemic antibiotics did not show a clinically significant effect [8,9,10]. On the other hand, the application of various topical antibiotics, including tetracycline and amoxicillin, provides limited efficacy in GTR treatment, including decreased bacterial contamination and increased clinical adherence [11,12,13,14]. In addition, several in vitro and in vivo studies have reported that drug-releasing membranes containing chlorhexidine digluconate solution or metronidazole prevent or delay bacterial adhesion to the membrane [4,15]. Nevertheless, as mentioned above, it has been reported that in ARP, the open membrane technique with exposure of the barrier membrane provides equivalent alveolar crest preservation compared to primary soft tissue closure [16,17], and it is unlikely that the bacterial colony formation on the membrane will have a negative effect on the clinical outcome of ARP. However, in all periodontal procedures, whether nonsurgical or surgical, poor plaque control is clearly detrimental to short-term and long-term periodontal tissue healing and health maintenance, including residual periodontal pockets, attachment loss, alveolar bone resorption, tooth loss, and peri-implant disease, and ARP would be no exception [18,19,20,21,22,23,24,25]. Therefore, antibiotics and antiseptics should only be used as adjuncts, and it is most important to first maintain a high level of oral hygiene throughout the entire treatment process.

Toxic substances such as nicotine and carbon monoxide in cigarette smoke are thought to adversely affect the clinical outcome of periodontal tissue regenerative therapy because they reduce microcirculation and blood flow in periodontal tissue [26,27] and disrupt the immune and inflammatory response systems [28,29]. Tonetti et al. [30] reported that cigarette smoking was associated with a decreased healing response in submarginal defects treated by GTR with a nonabsorbable ePTFE membrane. Although the rate of tissue gain at membrane removal between smokers and nonsmokers was not significantly different, the attachment level gained by smokers one year after treatment was significantly less. On the other hand, Zitzmann [31] evaluated various factors involved in the outcome of GBR treatment in a retrospective, clinical study and reported, based on multivariate regression analysis, that there was no clear evidence for the risk of smoking. Therefore, although few studies have investigated the direct effects of smoking on alveolar bone regeneration, the current scientific evidence does not show consistent results. It is presumed that Schwarz et al. [24] determined that there is no conclusive evidence for smoking as a risk factor for peri-implantitis in the new international classification of periodontal disease of the American Academy of Periodontology and European Federation of Periodontology due to differences in the definition of smoking and other background factors such as a history of periodontitis between studies. Nevertheless, several studies have shown that early implant loss is more common in smokers [32,33] and that smoking may increase the risk of developing peri-implantitis [34]. In addition, a growing number of studies strongly support that smoking is a significant environmental risk factor for the pathological progression of periodontal disease and treatment responsiveness [35,36,37,38,39]. Therefore, even if the evidence is equivocal, it should be recommended that all smoking patients at least follow a smoking cessation protocol or quit smoking altogether whenever possible.

With regard to diabetes mellitus, it is also believed that the impaired function of polymorphonuclear leucocytes [40,41], abnormal collagen metabolism [42], and delayed wound healing due to impaired microcirculation [43] resulting from hyperglycemia and insulin resistance may inhibit periodontal tissue regeneration [44,45]. Animal studies have shown that experimental diabetes mellitus interferes with the therapeutic efficacy of periodontal tissue regenerative therapy and GBR procedures [46,47]. However, in human clinical studies, the effects of diabetes mellitus on surgical procedures using barrier membranes and bone graft material, such as GTR and GBR procedures, as well as ARP, have not been reported at all to date. With regard to periodontal tissue regenerative therapy, a prospective study by Mizutani et al. [48] showed that minimally invasive surgery combined with EMD resulted in clinically significant attachment gain and bone filling, with similar levels in both diabetic and non-diabetic groups. Of note, because the glycemic status of the diabetic group subjects in this study was well controlled (mean HbA1c = 6.82), there is a high potential for negative periodontal tissue regeneration results for periodontal tissue parameters and bone regeneration in poorly controlled, severely diabetic patients. As with smoking, there does not appear to be sufficient evidence to conclude that diabetes mellitus is a risk factor affecting the long-term prognosis of implants or peri-implantitis.

## 8. Concluding Remarks

Spontaneous healing after tooth extraction is always accompanied by significant, three-dimensional shrinkage of the alveolar ridge, which can compromise proper implant placement and prosthetic esthetics. Therefore, it is advisable to perform ARP strategically by anticipating the potential for future implant treatment during the treatment planning phase prior to tooth extraction. In doing so, additional ridge augmentation in bone and soft tissue may be avoided, minimizing cost and invasiveness. Even in immediate implant placement, when used in combination with grafting materials, the alveolar ridge retraction can be reduced, which can be viewed as ARP in a broad sense.

However, there is considerable heterogeneity in the barrier membranes and bone grafting materials used for ARP, and the selection criteria do not currently appear to be supported by sufficient scientific evidence. Regarding the type of barrier membrane, free gingival grafts, connective tissue grafts, alternative materials such as collagen and synthetic polymeric membranes, and d-PTFE membranes are effective, and the decision regarding which membrane to use tends to be made at the discretion of the operators, depending on the need to obtain keratinized tissue width and thickness, the bone defect configuration of the extraction socket, and flap design. The rationale for selecting bone graft material is even more ambiguous and varies widely depending on the clinician’s and patient’s preference, economic considerations, and cultural and educational background.

A recent Cochrane review by Atieh et al. [23] reported no statistically significant differences between various bone graft materials or membranes used for ARP. However, the studies included in the meta-analysis compared only the amount of dimensional change on cone-beam computed tomography and were, thus, unable to assess the acceptability of implant placement, esthetic gain, and subsequent clinical long-term outcomes. Nevertheless, the clinical significance of ARP seems to be widely accepted because, even if the preserved amount is 1 mm of bone width or bone height diameter with no statistically significant difference, that 1 mm dimension can significantly alter implant placement options.

Consequently, in view of the current lack of sufficient high-quality clinical research, we have determined that the creation of flowcharts and decision trees to assist in treatment planning is premature. To achieve this objective in the future, there will need to be an increasing number of larger, more tightly controlled, randomized, controlled studies with study designs to compare different materials for ARP as simply as possible.

## Data Availability

Not applicable.

## References

[1] Atwood D.A. (1971). Reduction of residual ridges: A major oral disease entity. J. Prosthet. Dent..

[2] Araujo M.G., Lindhe J. (2005). Dimensional ridge alterations following tooth extraction. An experimental study in the dog. J. Clin. Periodontol..

[3] Pietrokovski J., Massler M. (1967). Alveolar ridge resorption following tooth extraction. J. Prosthet. Dent..

[4] Schropp L., Wenzel A., Kostopoulos L., Karring T. (2003). Bone healing and soft tissue contour changes following single-tooth extraction: A clinical and radiographic 12-month prospective study. Int. J. Periodontics Restor. Dent..

[5] John V., De Poi R., Blanchard S. (2007). Socket preservation as a precursor of future implant placement: Review of the literature and case reports. Compend. Contin. Educ. Dent..

[6] Chatzopoulos G.S., Koidou V.P., Sonnenberger M., Johnson D., Chu H., Wolff L.F. (2022). Postextraction ridge preservation by using dense PTFE membranes: A systematic review and meta-analysis. J. Prosthet. Dent..

[7] Avila-Ortiz G., Chambrone L., Vignoletti F. (2019). Effect of alveolar ridge preservation interventions following tooth extraction: A systematic review and meta-analysis. J. Clin. Periodontol..

[8] Avila-Ortiz G., Elangovan S., Kramer K.W., Blanchette D., Dawson D.V. (2014). Effect of alveolar ridge preservation after tooth extraction: A systematic review and meta-analysis. J. Dent. Res..

[9] Ashman A., Bruins P. (1982). A new immediate hard tissue replacement (HTR)TM for bone in the oral cavity. J. Oral Implantol..

[10] Avila-Ortiz G., Gubler M., Romero-Bustillos M., Nicholas C.L., Zimmerman M.B., Barwacz C.A. (2020). Efficacy of Alveolar Ridge Preservation: A Randomized Controlled Trial. J. Dent. Res..

[11] Chavda S., Levin L. (2018). Human Studies of Vertical and Horizontal Alveolar Ridge Augmentation Comparing Different Types of Bone Graft Materials: A Systematic Review. J. Oral Implantol..

[12] Casarez-Quintana A., Mealey B.L., Kotsakis G., Palaiologou A. (2022). Comparing the histological assessment following ridge preservation using a composite bovine-derived xenograft versus an alloplast hydroxyapatite-sugar cross-linked collagen matrix. J. Periodontol..

[13] Myeroff C., Archdeacon M. (2011). Autogenous bone graft: Donor sites and techniques. J. Bone Jt. Surg. Am..

[14] Lazzara R.J. (1989). Immediate implant placement into extraction sites: Surgical and restorative advantages. Int. J. Periodontics Restor. Dent..

[15] Becker W., Becker B.E. (1990). Guided tissue regeneration for implants placed into extraction sockets and for implant dehiscences: Surgical techniques and case report. Int. J. Periodontics Restor. Dent..

[16] Lekovic V., Klokkevold P.R., Camargo P.M., Kenney E.B., Nedic M., Weinlaender M. (1998). Evaluation of periosteal membranes and coronally positioned flaps in the treatment of Class II furcation defects: A comparative clinical study in humans. J. Periodontol..

[17] Stimmelmayr M., Guth J.F., Iglhaut G., Beuer F. (2012). Preservation of the ridge and sealing of the socket with a combination epithelialised and subepithelial connective tissue graft for management of defects in the buccal bone before insertion of implants: A case series. Br. J. Oral Maxillofac. Surg..

[18] Bartee B.K. (1995). The use of high-density polytetrafluoroethylene membrane to treat osseous defects: Clinical reports. Implant Dent..

[19] Oguic M., Candrlic M., Tomas M., Vidakovic B., Blaskovic M., Jerbic Radetic A.T., Zoricic Cvek S., Kuis D., Cvijanovic Peloza O. (2023). Osteogenic Potential of Autologous Dentin Graft Compared with Bovine Xenograft Mixed with Autologous Bone in the Esthetic Zone: Radiographic, Histologic and Immunohistochemical Evaluation. Int. J. Mol. Sci..

[20] Sapoznikov L., Haim D., Zavan B., Scortecci G., Humphrey M.F. (2023). A novel porcine dentin-derived bone graft material provides effective site stability for implant placement after tooth extraction: A randomized controlled clinical trial. Clin. Oral Investig..

[21] Kutkut A., Andreana S., Kim H.L., Monaco E. (2012). Extraction socket preservation graft before implant placement with calcium sulfate hemihydrate and platelet-rich plasma: A clinical and histomorphometric study in humans. J. Periodontol..

[22] Brahma Prasad Chary N.O., Raju M.S., Suresh Sajjan M.C., Gottumukkala S.N., Manyam R. (2021). Comparison of quality of bone and insertion torque values of early implants placed at 6 and 8 weeks in sockets preserved with advanced platelet-rich fibrin: A randomized controlled trial. J. Indian Prosthodont. Soc..

[23] Atieh M.A., Alsabeeha N.H., Payne A.G., Ali S., Faggion C.M.J., Esposito M. (2021). Interventions for replacing missing teeth: Alveolar ridge preservation techniques for dental implant site development. Cochrane Database Syst. Rev..

[24] Tan W.L., Wong T.L., Wong M.C., Lang N.P. (2012). A systematic review of post-extractional alveolar hard and soft tissue dimensional changes in humans. Clin. Oral Implant. Res..

[25] Johnson K. (1969). A study of the dimensional changes occurring in the maxilla following tooth extraction. Aust. Dent. J..

[26] Lam R.V. (1960). Contour changes of the alveolar processes following extractions. J. Prosthet. Dent..

[27] Araujo M.G., Silva C.O., Misawa M., Sukekava F. (2015). Alveolar socket healing: What can we learn?. Periodontol 2000.

[28] Chappuis V., Engel O., Shahim K., Reyes M., Katsaros C., Buser D. (2015). Soft Tissue Alterations in Esthetic Postextraction Sites: A 3-Dimensional Analysis. J. Dent. Res..

[29] Chappuis V., Engel O., Reyes M., Shahim K., Nolte L.P., Buser D. (2013). Ridge alterations post-extraction in the esthetic zone: A 3D analysis with CBCT. J. Dent. Res..

[30] Misawa M., Lindhe J., Araujo M.G. (2016). The alveolar process following single-tooth extraction: A study of maxillary incisor and premolar sites in man. Clin. Oral Implant. Res..

[31] Schulte W., Kleineikenscheidt H., Lindner K., Schareyka R., Heimke G., Gerlach C., Hardegg W. (1978). Animal experiments on the question of healing around the Tubingen immediate implant. Dtsch. Zahnarztl. Z..

[32] Barzilay I., Graser G.N., Iranpour B., Natiella J.R. (1991). Immediate implantation of a pure titanium implant into an extraction socket: Report of a pilot procedure. Int. J. Oral Maxillofac. Implant..

[33] Lang N.P., Bragger U., Hammerle C.H., Sutter F. (1994). Immediate transmucosal implants using the principle of guided tissue regeneration. I. Rationale, clinical procedures and 30-month results. Clin. Oral Implant. Res..

[34] Denissen H.W., Kalk W., Veldhuis H.A., van Waas M.A. (1993). Anatomic consideration for preventive implantation. Int. J. Oral Maxillofac. Implant..

[35] Paolantonio M., Dolci M., Scarano A., d’Archivio D., di Placido G., Tumini V., Piattelli A. (2001). Immediate implantation in fresh extraction sockets. A controlled clinical and histological study in man. J. Periodontol..

[36] Watzek G., Haider R., Mensdorff-Pouilly N., Haas R. (1995). Immediate and delayed implantation for complete restoration of the jaw following extraction of all residual teeth: A retrospective study comparing different types of serial immediate implantation. Int. J. Oral Maxillofac. Implant..

[37] Araujo M.G., Sukekava F., Wennstrom J.L., Lindhe J. (2006). Tissue modeling following implant placement in fresh extraction sockets. Clin. Oral Implant. Res..

[38] Botticelli D., Berglundh T., Lindhe J. (2004). Hard-tissue alterations following immediate implant placement in extraction sites. J. Clin. Periodontol..

[39] Sanz M., Cecchinato D., Ferrus J., Pjetursson E.B., Lang N.P., Lindhe J. (2010). A prospective, randomized-controlled clinical trial to evaluate bone preservation using implants with different geometry placed into extraction sockets in the maxilla. Clin. Oral Implant. Res..

[40] Araujo M.G., Sukekava F., Wennstrom J.L., Lindhe J. (2005). Ridge alterations following implant placement in fresh extraction sockets: An experimental study in the dog. J. Clin. Periodontol..

[41] Araujo M.G., Wennstrom J.L., Lindhe J. (2006). Modeling of the buccal and lingual bone walls of fresh extraction sites following implant installation. Clin. Oral Implant. Res..

[42] Ferrus J., Cecchinato D., Pjetursson E.B., Lang N.P., Sanz M., Lindhe J. (2010). Factors influencing ridge alterations following immediate implant placement into extraction sockets. Clin. Oral Implant. Res..

[43] Matarasso S., Salvi G.E., Iorio Siciliano V., Cafiero C., Blasi A., Lang N.P. (2009). Dimensional ridge alterations following immediate implant placement in molar extraction sites: A six-month prospective cohort study with surgical re-entry. Clin. Oral Implant. Res..

[44] Chen S.T., Darby I.B., Reynolds E.C. (2007). A prospective clinical study of non-submerged immediate implants: Clinical outcomes and esthetic results. Clin. Oral Implant. Res..

[45] Hammerle C.H., Araujo M.G., Simion M., Osteology Consensus G. (2012). Evidence-based knowledge on the biology and treatment of extraction sockets. Clin. Oral Implant. Res..

[46] Artzi Z., Nemcovsky C.E. (1998). The application of deproteinized bovine bone mineral for ridge preservation prior to implantation. Clinical and histological observations in a case report. J. Periodontol..

[47] De Risi V., Clementini M., Vittorini G., Mannocci A., De Sanctis M. (2015). Alveolar ridge preservation techniques: A systematic review and meta-analysis of histological and histomorphometrical data. Clin. Oral Implant. Res..

[48] Horvath A., Mardas N., Mezzomo L.A., Needleman I.G., Donos N. (2013). Alveolar ridge preservation. A systematic review. Clin. Oral Investig..

[49] Canellas J., Soares B.N., Ritto F.G., Vettore M.V., Vidigal Junior G.M., Fischer R.G., Medeiros P.J.D. (2021). What grafting materials produce greater alveolar ridge preservation after tooth extraction? A systematic review and network meta-analysis. J. Craniomaxillofac. Surg..

[50] Landsberg C.J. (1997). Socket seal surgery combined with immediate implant placement: A novel approach for single-tooth replacement. Int. J. Periodontics Restor. Dent..

[51] Landsberg C.J., Bichacho N. (1994). A modified surgical/prosthetic approach for optimal single implant supported crown. Part I—The socket seal surgery. Pract. Periodontics Aesthet. Dent..

[52] Jung R.E., Siegenthaler D.W., Hammerle C.H. (2004). Postextraction tissue management: A soft tissue punch technique. Int. J. Periodontics Restor. Dent..

[53] Stimmelmayr M., Allen E.P., Reichert T.E., Iglhaut G. (2010). Use of a combination epithelized-subepithelial connective tissue graft for closure and soft tissue augmentation of an extraction site following ridge preservation or implant placement: Description of a technique. Int. J. Periodontics Restor. Dent..

[54] Papace C., Busch C., Ristow O., Keweloh M., Hoffmann J., Mertens C. (2021). The effect of different soft-tissue management techniques for alveolar ridge preservation: A randomized controlled clinical trial. Int. J. Implant. Dent..

[55] Segnini B., Borges-Filho F.F., Nicoli L.G., Goncalves M., Marcantonio C., Oliveira G.J., Marcantonio E. (2021). Impact of soft tissue graft on the preservation of compromised sockets: A randomized controlled clinical pilot study. Acta Odontol. Latinoam..

[56] Chambrone L., Sukekava F., Araujo M.G., Pustiglioni F.E., Chambrone L.A., Lima L.A. (2010). Root-coverage procedures for the treatment of localized recession-type defects: A Cochrane systematic review. J. Periodontol..

[57] Oates T.W., Robinson M., Gunsolley J.C. (2003). Surgical therapies for the treatment of gingival recession. A systematic review. Ann. Periodontol..

[58] McGuire M.K., Scheyer E.T. (2014). Randomized, controlled clinical trial to evaluate a xenogeneic collagen matrix as an alternative to free gingival grafting for oral soft tissue augmentation. J. Periodontol..

[59] Lim H.C., Jung U.W., You H., Lee J.S. (2017). Randomized clinical trial of ridge preservation using porcine bone/cross-linked collagen vs. bovine bone/non-cross-linked collagen: Cone beam computed tomographic analysis. Clin. Oral Implant. Res..

[60] Barber H.D., Lignelli J., Smith B.M., Bartee B.K. (2007). Using a dense PTFE membrane without primary closure to achieve bone and tissue regeneration. J. Oral Maxillofac. Surg..

[61] Simion M., Baldoni M., Rossi P., Zaffe D. (1994). A comparative study of the effectiveness of e-PTFE membranes with and without early exposure during the healing period. Int. J. Periodontics Restor. Dent..

[62] Bartee B.K. (2001). Extraction site reconstruction for alveolar ridge preservation. Part 2: Membrane-assisted surgical technique. J. Oral Implantol..

[63] Bartee B.K. (1998). Evaluation of a new polytetrafluoroethylene guided tissue regeneration membrane in healing extraction sites. Compend. Contin. Educ. Dent..

[64] Arbab H., Greenwell H., Hill M., Morton D., Vidal R., Shumway B., Allan N.D. (2016). Ridge Preservation Comparing a Nonresorbable PTFE Membrane to a Resorbable Collagen Membrane: A Clinical and Histologic Study in Humans. Implant Dent..

[65] Sun D.J., Lim H.C., Lee D.W. (2019). Alveolar ridge preservation using an open membrane approach for sockets with bone deficiency: A randomized controlled clinical trial. Clin. Implant Dent. Relat. Res..

[66] Lee J., Lee J.B., Koo K.T., Seol Y.J., Lee Y.M. (2018). Flap Management in Alveolar Ridge Preservation: A Systematic Review and Meta-Analysis. Int. J. Oral Maxillofac. Implant..

[67] Engler-Hamm D., Cheung W.S., Yen A., Stark P.C., Griffin T. (2011). Ridge preservation using a composite bone graft and a bioabsorbable membrane with and without primary wound closure: A comparative clinical trial. J. Periodontol..

[68] Barone A., Borgia V., Covani U., Ricci M., Piattelli A., Iezzi G. (2015). Flap versus flapless procedure for ridge preservation in alveolar extraction sockets: A histological evaluation in a randomized clinical trial. Clin. Oral Implant. Res..

[69] Martins J.R., Wagner T.P., Vallim A.C., Konflanz W., Schwendicke F., Celeste R.K., Haas A.N. (2022). Comparison of the efficacy of different techniques to seal the alveolus during alveolar ridge preservation: Meta-regression and network meta-analysis. J. Clin. Periodontol..

[70] Brkovic B.M., Prasad H.S., Rohrer M.D., Konandreas G., Agrogiannis G., Antunovic D., Sandor G.K. (2012). Beta-tricalcium phosphate/type I collagen cones with or without a barrier membrane in human extraction socket healing: Clinical, histologic, histomorphometric, and immunohistochemical evaluation. Clin. Oral Investig..

[71] Lim H.C., Shin H.S., Cho I.W., Koo K.T., Park J.C. (2019). Ridge preservation in molar extraction sites with an open-healing approach: A randomized controlled clinical trial. J. Clin. Periodontol..

[72] Saito H., Couso-Queiruga E., Shiau H.J., Stuhr S., Prasad H., Allareddy T.V., Reynolds M.A., Avila-Ortiz G. (2021). Evaluation of poly lactic-co-glycolic acid-coated beta-tricalcium phosphate for alveolar ridge preservation: A multicenter randomized controlled trial. J. Periodontol..

[73] Jung R.E., Philipp A., Annen B.M., Signorelli L., Thoma D.S., Hammerle C.H., Attin T., Schmidlin P. (2013). Radiographic evaluation of different techniques for ridge preservation after tooth extraction: A randomized controlled clinical trial. J. Clin. Periodontol..

[74] Jamjoom A., Cohen R.E. (2015). Grafts for Ridge Preservation. J. Funct. Biomater..

[75] Goldberg V.M., Stevenson S. (1987). Natural history of autografts and allografts. Clin. Orthop. Relat. Res..

[76] Zhao R., Yang R., Cooper P.R., Khurshid Z., Shavandi A., Ratnayake J. (2021). Bone Grafts and Substitutes in Dentistry: A Review of Current Trends and Developments. Molecules.

[77] Dias R.R., Sehn F.P., de Santana Santos T., Silva E.R., Chaushu G., Xavier S.P. (2016). Corticocancellous fresh-frozen allograft bone blocks for augmenting atrophied posterior mandibles in humans. Clin. Oral Implant. Res..

[78] Quattlebaum J.B., Mellonig J.T., Hensel N.F. (1988). Antigenicity of freeze-dried cortical bone allograft in human periodontal osseous defects. J. Periodontol..

[79] The Committee on Research, Science and Therapy of the American Academy of Periodontology (2001). Position Paper; Tissue Banking of Bone Allografts Used in Periodontal Regeneration. J. Periodontol..

[80] Russell J., Scarborough N., Chesmel K. (1997). Re: Ability of commercial demineralized freeze-dried bone allograft to induce new bone formation (**1996**, *67*, 918–926). J. Periodontol..

[81] Mellonig J.T., Bowers G.M., Bailey R.C. (1981). Comparison of bone graft materials. Part I. New bone formation with autografts and allografts determined by Strontium-85. J. Periodontol..

[82] McAllister B.S., Margolin M.D., Cogan A.G., Buck D., Hollinger J.O., Lynch S.E. (1999). Eighteen-month radiographic and histologic evaluation of sinus grafting with anorganic bovine bone in the chimpanzee. Int. J. Oral Maxillofac. Implant..

[83] Sogal A., Tofe A.J. (1999). Risk assessment of bovine spongiform encephalopathy transmission through bone graft material derived from bovine bone used for dental applications. J. Periodontol..

[84] Shetty V., Han T.J. (1991). Alloplastic materials in reconstructive periodontal surgery. Dent. Clin. N. Am..

[85] Wang H., Li Y., Zuo Y., Li J., Ma S., Cheng L. (2007). Biocompatibility and osteogenesis of biomimetic nano-hydroxyapatite/polyamide composite scaffolds for bone tissue engineering. Biomaterials.

[86] Osborn J.F., Newesely H. (1980). The material science of calcium phosphate ceramics. Biomaterials.

[87] Klein C.P., Driessen A.A., de Groot K., van den Hooff A. (1983). Biodegradation behavior of various calcium phosphate materials in bone tissue. J. Biomed. Mater. Res..

[88] Rabalais M.L., Yukna R.A., Mayer E.T. (1981). Evaluation of durapatite ceramic as an alloplastic implant in periodontal osseous defects. I. Initial six-month results. J. Periodontol..

[89] Schepers E., de Clercq M., Ducheyne P., Kempeneers R. (1991). Bioactive glass particulate material as a filler for bone lesions. J. Oral Rehabil..

[90] Skallevold H.E., Rokaya D., Khurshid Z., Zafar M.S. (2019). Bioactive Glass Applications in Dentistry. Int. J. Mol. Sci..

[91] Carvalho S.M., Oliveira A.A., Jardim C.A., Melo C.B., Gomes D.A., de Fatima Leite M., Pereira M.M. (2012). Characterization and induction of cementoblast cell proliferation by bioactive glass nanoparticles. J. Tissue Eng. Regen. Med..

[92] Lee J.H., Jeong S.N. (2020). Effect of enamel matrix derivative on alveolar ridge preservation in the posterior maxilla: A randomized controlled clinical trial. Clin. Implant Dent. Relat. Res..

[93] Mercado F., Vaquette C., Hamlet S., Ivanovski S. (2021). Enamel matrix derivative promotes new bone formation in xenograft assisted maxillary anterior ridge preservation-A randomized controlled clinical trial. Clin. Oral Implant. Res..

[94] Eeckhout C., Ackerman J., Glibert M., Cosyn J. (2022). A randomized controlled trial evaluating hyaluronic acid gel as wound healing agent in alveolar ridge preservation. J. Clin. Periodontol..

[95] Barone A., Orlando B., Cingano L., Marconcini S., Derchi G., Covani U. (2012). A randomized clinical trial to evaluate and compare implants placed in augmented versus non-augmented extraction sockets: 3-year results. J. Periodontol..

[96] Festa V.M., Addabbo F., Laino L., Femiano F., Rullo R. (2013). Porcine-derived xenograft combined with a soft cortical membrane versus extraction alone for implant site development: A clinical study in humans. Clin. Implant Dent. Relat. Res..

[97] Cha J.K., Song Y.W., Park S.H., Jung R.E., Jung U.W., Thoma D.S. (2019). Alveolar ridge preservation in the posterior maxilla reduces vertical dimensional change: A randomized controlled clinical trial. Clin. Oral Implant. Res..

[98] Iorio-Siciliano V., Ramaglia L., Blasi A., Bucci P., Nuzzolo P., Riccitiello F., Nicolo M. (2020). Dimensional changes following alveolar ridge preservation in the posterior area using bovine-derived xenografts and collagen membrane compared to spontaneous healing: A 6-month randomized controlled clinical trial. Clin. Oral Investig..

[99] Santana R., Gyurko R., Kanasi E., Xu W.P., Dibart S. (2019). Synthetic polymeric barrier membrane associated with blood coagulum, human allograft, or bovine bone substitute for ridge preservation: A randomized, controlled, clinical and histological trial. Int. J. Oral Maxillofac. Surg..

[100] Scheyer E.T., Heard R., Janakievski J., Mandelaris G., Nevins M.L., Pickering S.R., Richardson C.R., Pope B., Toback G., Velasquez D. (2016). A randomized, controlled, multicentre clinical trial of post-extraction alveolar ridge preservation. J. Clin. Periodontol..

[101] Serrano Mendez C.A., Lang N.P., Caneva M., Ramirez Lemus G., Mora Solano G., Botticelli D. (2017). Comparison of allografts and xenografts used for alveolar ridge preservation. A clinical and histomorphometric RCT in humans. Clin. Implant Dent. Relat. Res..

[102] Chisci G., Hatia A., Chisci E., Chisci D., Gennaro P., Gabriele G. (2023). Socket Preservation after Tooth Extraction: Particulate Autologous Bone vs. Deproteinized Bovine Bone. Bioengineering.

[103] Shim J.Y., Lee Y., Lim J.H., Jin M.U., Lee J.M., Suh J.Y., Kim Y.G. (2018). Comparative Evaluation of Recombinant Human Bone Morphogenetic Protein-2/Hydroxyapatite and Bovine Bone for New Bone Formation in Alveolar Ridge Preservation. Implant Dent..

[104] Bonta H., Galli F.G., Gualtieri A., Renou S., Caride F. (2022). The Effect of an Alloplastic Bone Substitute and Enamel Matrix Derivative on the Preservation of Single Anterior Extraction Sockets: A Histologic Study in Humans. Int. J. Periodontics Restor. Dent..

[105] Mayer Y., Zigdon-Giladi H., Machtei E.E. (2016). Ridge Preservation Using Composite Alloplastic Materials: A Randomized Control Clinical and Histological Study in Humans. Clin. Implant Dent. Relat. Res..

[106] Noronha Oliveira M., Rau L.H., Marodin A., Correa M., Correa L.R., Aragones A., Magini R.S. (2017). Ridge Preservation After Maxillary Third Molar Extraction Using 30% Porosity PLGA/HA/beta-TCP Scaffolds With and Without Simvastatin: A Pilot Randomized Controlled Clinical Trial. Implant Dent..

[107] Cortellini P. (2012). Minimally invasive surgical techniques in periodontal regeneration. J. Evid. Based Dent. Pract..

[108] Becker W., Clokie C., Sennerby L., Urist M.R., Becker B.E. (1998). Histologic findings after implantation and evaluation of different grafting materials and titanium micro screws into extraction sockets: Case reports. J. Periodontol..

[109] Carmagnola D., Adriaens P., Berglundh T. (2003). Healing of human extraction sockets filled with Bio-Oss. Clin. Oral Implant. Res..

